# Quality and reliability assessment of systemic lupus erythematosus related health information on short video platforms

**DOI:** 10.3389/fdgth.2026.1875817

**Published:** 2026-08-13

**Authors:** Lingling Gao, Yujie Yang, Yuchen Wang, Kunling Wang, Wanqing Li, Hongrui Wen, Yunan Zhang, Xiangfeng Li, Wei Bai

**Affiliations:** 1 Peking Union Medical College Hospital (CAMS), Beijing, China; 2 Chinese Academy of Medical Sciences and Peking Union Medical College, Beijing, China

**Keywords:** health information, quality assessment, short video, social media, systemic lupus erythematosus

## Abstract

**Background:**

Systemic lupus erythematosus (SLE) is a chronic autoimmune disease involving multiple organ systems. Patient education is an essential component of disease management. With the rapid development of digital media, short-video platforms have become increasingly important health information sources for SLE patients. Therefore, the quality of health information disseminated through these platforms warrants attention. However, evidence regarding the quality of SLE-related health information on short-video platforms remains limited.

**Methods:**

On September 18, 2025, SLE-related videos were retrieved from TikTok, Rednote (Xiaohongshu), and Bilibili. We evaluated the videos' reliability using the DISCERN instrument, their overall quality using the Global Quality Scale (GQS), and their understandability and actionability using the Patient Education Materials Assessment Tool (PEMAT-U/A).

**Results:**

A total of 276 SLE-related videos from three platforms were analyzed. Significant differences in video quality and reliability were observed among platforms (*p* < 0.001). Specifically, videos on Rednote exhibited the highest quality, while TikTok videos demonstrated the lowest quality. Videos produced by online health science communicators and certified medical professionals had significantly higher DISCERN and PEMAT-U scores than patient-generated videos (*p* < 0.05). Correlation analysis indicated that video engagement metrics were positively associated with quality and reliability on Bilibili (*p* < 0.01), whereas a negative correlation was found on TikTok and Rednote (e.g., DISCERN vs. likes on TikTok: *r* = −0.327, *p* = 0.001; GQS vs. collections on Rednote: *r* = −0.365, *p* = 0.006). Regarding the relationship between quality and engagement, DISCERN and GQS, and PEMAT-U scores on Bilibili were positively correlated with engagement metrics (*p* < 0.01). In contrast, several inverse associations were identified on TikTok and Rednote, including negative correlations between DISCERN scores and likes on TikTok (*r* = −0.327, *p* = 0.001) and between GQS scores and collections on Rednote (*r* = −0.365, *p* = 0.006).

**Conclusion:**

The quality and reliability of SLE-related videos vary significantly across platforms and are generally suboptimal. The relationship between video quality and user engagement is platform- and measure-specific, suggesting higher-quality health information does not always translate into greater user engagement. Enhancing reliable health information requires coordinated efforts to improve both content quality and audience engagement.

## Introduction

Systemic lupus erythematosus (SLE) is a multisystem chronic autoimmune disorder with incompletely understood pathogenesis, modulated by genetic, hormonal, and infectious triggers (1–3). Its global prevalence is increasing, up to 43.7 per 100,000 population (4). The disease disproportionately affects women of childbearing age, with a male-to-female incidence ratio of roughly 1:9 (5). SLE manifests heterogeneously with common symptoms including fever, cutaneous rash, and arthralgia; advanced or severe disease may lead to irreversible renal, cardiac, and other end-organ damage. Clinically, SLE is difficult to treat and prone to frequent relapse (6, 7). As a chronic illness, SLE relies on long-term standardized self-management, placing patients at the center of disease control. Accordingly, targeted patient education and public outreach regarding SLE are essential to optimize overall disease management.

The rapid growth of digital media has profoundly reshaped how a substantial portion of the population seeks health information. It is estimated that 80% of internet users search for medical information online before receiving healthcare services (8). Video-sharing platforms have become a primary channel for health communication: YouTube, with over 2 billion monthly users, saw a 75% increase in health-related video consumption compared to pre-pandemic levels (9). Short-video platforms, in particular, offer notable advantages—easy accessibility, high interactivity, diverse presentation formats, and swift dissemination—making them powerful tools for health education (10–12). Nevertheless, the effective comprehension of such content hinges on individual health literacy. Leading health organizations, including the National Institutes of Health, the American Medical Association, and the U.S. Department of Health and Human Services, recommend that internet-based patient education materials be written at or below a sixth-grade reading level (13). Despite these recommendations, the actual quality of health information on short-video platforms frequently falls short, largely due to their open nature and the lack of professional content review (14). This results in considerable disparities in reliability and accuracy. For example, an evaluation of short videos on dietary recommendations for inflammatory bowel disease found that overall quality was inadequate and that substantial misleading information was present (15). Ultimately, while high-quality videos can enhance public understanding of complex health topics, low-quality videos may misdirect viewers and undermine the goals of public health education (16).

For individuals with chronic conditions, the knowledge gained from online sources can significantly influence clinical decisions and treatment outcomes (17). SLE patients, in particular, show a marked inclination to seek health information through online channels, including social media (18). This reliance has been further intensified by the digital transformation of healthcare information, with evaluating online video content a matter of direct clinical importance. Although video quality across platforms has been assessed for several diseases (19–21), research specifically focused on SLE remains scarce. Notably, there is a lack of studies that compare the quality of SLE-related health information across different short-video platforms.

TikTok (specifically the Chinese mainland version, known as Douyin), Bilibili and Rednote are the three dominant short-video platforms in China, each hosting hundreds of millions of monthly active users (22, 23). These platforms are free and user-friendly, and foster high interactivity through features such as likes, shares, comments, and bullet comments (23). Their diverse presentation styles further facilitate the public's acceptance of health information (23).

Although previous studies have evaluated video health information on these platforms for various diseases (19–21), systematic cross-platform comparisons focusing specifically on SLE remain limited (16–18). To address this research gap, the present study adopted the DISCERN tool, the Global Quality Scale (GQS), and the Patient Education Materials Assessment Tool for Audiovisual materials (PEMAT-A/V) to comprehensively assess the reliability, overall quality, and usability of SLE-related health videos published on TikTok, Rednote, and Bilibili. The findings aim to provide targeted recommendations for optimizing the quality of short-video health information and improving public health education regarding SLE.

## Methods

### Video retrieval strategy

This study adopted a cross-sectional design. On September 18, 2025, to minimize bias arising from personal search habits, we utilized newly registered accounts on TikTok (specifically the Chinese mainland version, known as Douyin), Rednote, and Bilibili to searches for the terms “系统性红斑狼疮” (systemic lupus erythematosus) or “红斑狼疮” or “狼疮” or “SLE”. All retrievable videos obtained using these four predefined search terms on each platform were collected, and no predefined number of top-ranked videos was selected. The search results from the four terms were combined within each platform, and duplicate videos were identified and removed during the screening process. Videos published after September 18, 2025 were excluded. The remaining videos were screened according to the following exclusion criteria: (1) videos not related to SLE; (2) videos lacking substantial health education content; (3) videos in pure English without Chinese subtitles; (4) videos with no sound; (5) unplayable videos; and (6) advertisement videos.

### Video data collection

After screening, we ultimately included a total of 276 videos from three platforms. We recorded fundamental information for each video, which encompasses the publisher's information, interaction and dissemination metrics, and the video's category. The essential information for each video includes the title, publishing platform, video link/URL, publishing time, and duration. The publisher's information comprises the publisher ID and video source. We categorized the video sources into five groups: (1) medical institutions, (2) certified medical professionals, (3) patients, (4) online science communicators, and (5) news organizations. Interaction and dissemination metrics include likes, collections, shares, and comments. Furthermore, we classified the video categories into five types: (1) disease knowledge, (2) symptoms, (3) experience sharing, (4) treatments, and (5) daily care and emotional support.

### Video quality assessment

We utilized DISCERN, GQS, and PEMAT to evaluate the quality and reliability of the videos. DISCERN is a widely recognized and validated tool for assessing the quality of healthcare information (24). It comprises 16 items categorized into three dimensions: items 1–9 evaluate the reliability of the information, items 10–15 provide specific details regarding treatment options, and item 16 offers an overall assessment. Each item is rated on a 5-point Likert scale, ranging from “completely disagree” (1) to “completely agree” (5). Previous studies have demonstrated that its overall Cronbach's *α* coefficient is 0.93, indicating stable and reliable internal consistency (25). In this study, all 16 items of the DISCERN instrument were rated for each included video. If a video lacked content corresponding to a specific item (e.g., statements about information sources or uncertainties in evidence), that item was assigned a score of 1 (“completely disagree”). This scoring approach adheres to official DISCERN guidelines, which specify that omission of such relevant information represents an inherent flaw in information quality. We employed the GQS to gauge the overall quality of the videos on a 1–5 scale, where higher scores indicate superior video quality. GQS is straightforward to use, concise, and has been extensively applied in related research (26, 27). Furthermore, this study used the PEMAT-A/V, the version specifically designed for audiovisual materials, making it appropriate for video-based health information. Empirical evidence has demonstrated that this scale exhibits good internal consistency: Cronbach's *α* coefficient is 0.77 for the Understandability subscale and 0.74 for the Actionability subscale (28). The tool comprises 17 items across the comprehensibility and actionability domains, each scored on a 0 (disagree) to 1 (agree) scale or N/A (not applicable). To accommodate the short-video format, operational criteria were prespecified for PEMAT-A/V items that rely on traditional text structures. For instance, “clear headings” were identified through prominent on-screen text, and “summarizing key points” through closing captions or final spoken takeaways. These criteria were documented in a rater guide and pilot-tested, with no modification to the original items or scoring scale.

From October 13 to November 13, 2025, two independent researchers, GL and WY, conducted the scoring. Both researchers had received systematic training in the use of the evaluation tool and possessed clinical experience in rheumatology and immunology. The average of their scores was utilized as the final result. In cases of significant disagreement between the two raters, a third researcher, YY, performed the final assessment. To evaluate the reliability and objectivity of the scoring criteria, this study implemented a scorer consistency test. The intraclass correlation coefficient (ICC) was used to assess interrater and intrarater reliability. ICC values were interpreted as follows: <0.5 = poor, 0.5–0.75 = moderate, 0.75–0.9 = good, and >0.9 = excellent. Interrater reliability was good for all measures: DISCERN score [ICC = 0.82, 95% CI (0.78, 0.91)], GQS score [ICC = 0.78, 95% CI (0.71, 0.84)], PEMAT-U scores [ICC = 0.84, 95% CI (0.78, 0.87)], and PEMAT-A scores [ICC = 0.85, 95% CI (0.81, 0.88)].

### Ethical considerations

This study does not require an ethical review, as it does not involve clinical data, human samples, experimental animals, or personal privacy concerns. All information utilized in this research is sourced from publicly available videos on platforms such as TikTok, Rednote, and Bilibili.

### Statistical analysis

Statistical analyses were conducted using SPSS (version 26.0) and GraphPad Prism (version 10.01). Categorical variables were reported as frequencies and percentages. Categorical variables were reported as frequencies and percentages and compared using the chi-square test or Fisher's exact test, as appropriate. Normally distributed continuous variables were presented as means and standard deviations, while non-normally distributed continuous variables were described using medians and interquartile ranges. Differences among the three platforms were assessed using the Kruskal–Wallis H test, followed by Bonferroni-adjusted pairwise comparisons when significant differences were detected. Spearman's rank correlation analysis was performed to assess associations between variables. A two-sided *p*-value < 0.05 was considered statistically significant.

## Results

### General characteristics of videos

**Figure 1:**
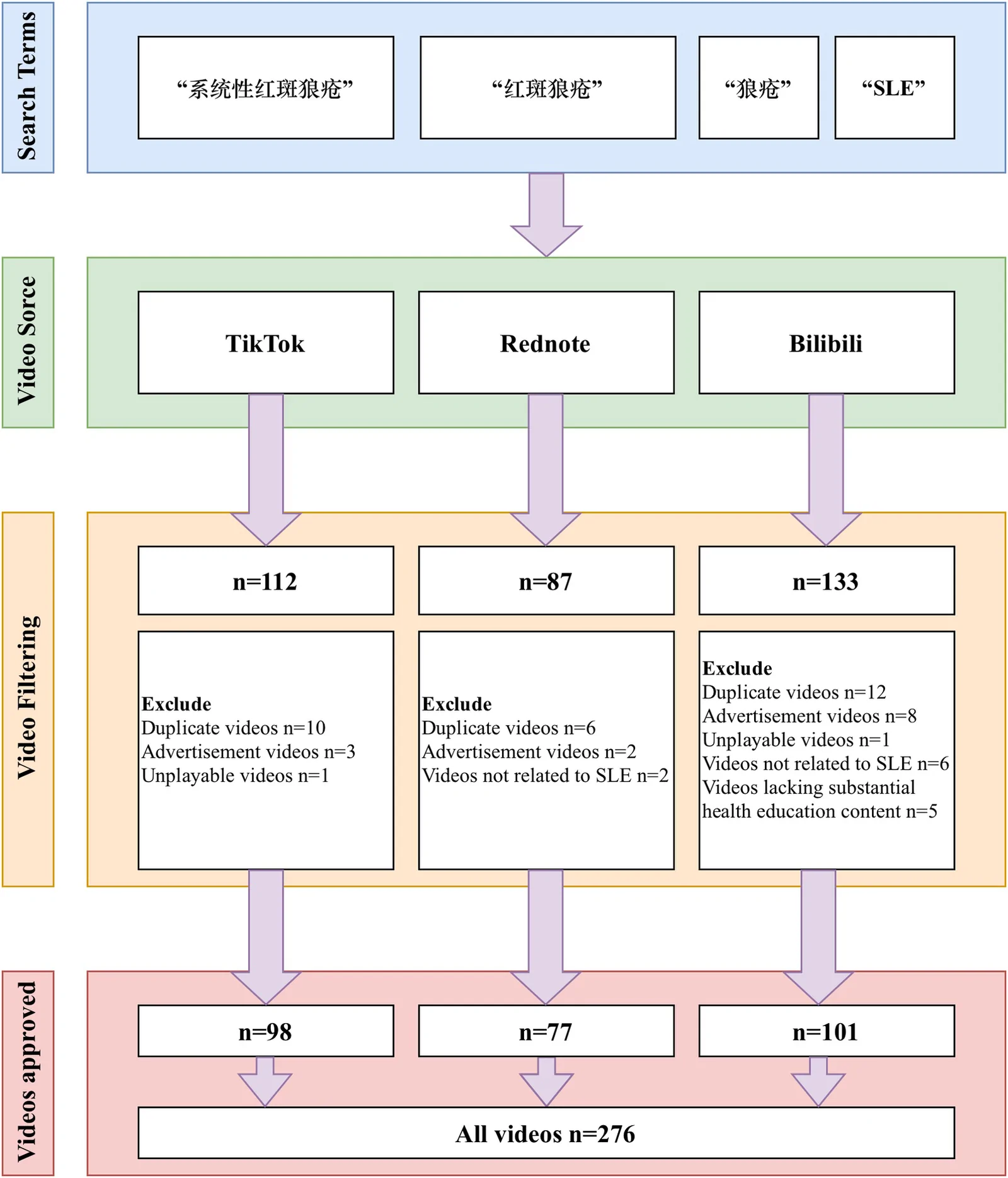
Flowchart of the study.

We collected all searchable videos from TikTok, Rednote, and Bilibili, sorting them by the number of likes in descending order. Subsequently, we eliminated unqualified videos based on established exclusion criteria. This process resulted in the removal of 14 videos from TikTok, 10 from Rednote, and 32 from Bilibili, leaving a total of 276 videos for further research and analysis. The final distribution includes 98 videos on TikTok (35.5%), 77 videos on Rednote (27.9%), and 101 videos on Bilibili (36.6%). Refer to Figure 1 for a detailed overview of the screening process.

### Video source and category

Table 1 presents the sources and categories of videos related to SLE. On the TikTok, Rednote, and Bilibili platforms, videos uploaded by certified medical professionals constituted the highest proportions, at 52%, 74%, and 58.4%, respectively. In terms of video categories, “disease knowledge” was the most prevalent across all platforms: TikTok (52%), Rednote (55.8%), and Bilibili (81.2%). Notably, neither “treatments” nor “daily care and emotional support” videos were found on the Bilibili platform.

**Table 1:** Characteristics and scoring results of videos concerning SLE.

Parameters	TikTok (*N* = 98)	Rednote (*N* = 77)	Bilibili (*N* = 101)	*p*-value
Duration (seconds), median (range)	96 (6–644)	92 (27–675)	41 (8–4,850)	<0.001
Likes, median (range)	1,640 (9–115,143)	25 (1–2,510)	2 (0–23,000)	<0.001
Collections, median (range)	505 (2–17,922)	3 (0–783)	1 (1–22,000)	<0.001
Comments, median (range)	356.5 (0–13,770)	3 (0–783)	0 (0–3,186)	<0.001
Shares, median (range)	551.5 (1–46,869)	8 (0–613)	0 (0–2,005)	<0.001
Video source				
Medical Institutions (*n*, %)	7 (7.1)	3 (3.9)	12 (11.9)	<0.001
Certified medical professionals (*n*, %)	51 (52)	57 (74)	59 (58.4)	<0.001
Patients (*n*, %)	22 (22.4)	13 (16.9)	16 (15.8)	<0.001
Online science communicators (*n*, %)	4 (4.1)	4 (5.2)	8 (7.9)	<0.001
News organizations (*n*, %)	14 (14.3)	0 (0)	6 (5.9)	<0.001
Video category				
Disease knowledge (*n*, %)	51 (52)	43 (55.8)	82 (81.2)	<0.005
Symptoms (*n*, %)	10 (10.2)	13 (16.9)	43 (42.6)	<0.005
Experience sharing (*n*, %)	23 (23.5)	12 (15.6)	48 (47.5)	<0.005
Treatments (*n*, %)	14 (14.3)	9 (11.7)	0 (0)	<0.005
Daily care and emotional support (*n*, %)	13 (13.3)	12 (15.6)	0(0)	<0.005

Data were presented as median (range) for continuous variables and *n* (%) for categorical variables. Continuous variables were compared using the Kruskal–Wallis H test. Categorical variables were compared using the chi-square test. For continuous variables and video source categories (mutually exclusive), *p*-values were unadjusted. For video category variables (non-mutually exclusive, 5 categories tested), Bonferroni correction was applied by multiplying the original *p*-values by 5; the *p*-values shown in the table for these comparisons are adjusted values (all original *p* < 0.001, adjusted *p* < 0.005).

### Video quality and reliability

Table 2 presents a comparative analysis of video quality scores, namely DISCERN, GQS, and PEMAT, across the TikTok, Rednote, and Bilibili platforms. Notably, Rednote videos achieved the highest scores in all three metrics. Bilibili's scores were moderate, while TikTok recorded the lowest scores. Statistical analysis revealed significant differences in all metrics among the three platforms (*p* < 0.001). However, no significant difference was observed in GQS scores between TikTok and Bilibili (*p* = 1.000). Additionally, neither Rednote nor Bilibili exhibited substantial differences in their PEMAT-U (the Patient Education Materials Assessment Tool-Understandability) scores (*p* = 0.196).

**Table 2:** Comparison of video quality and reliability scores among TikTok, Rednote and Bilibili platforms.

Categories	DISCERN	GQS	PEMAT-U	PEMAT-A
TikTok	32.5 (28.5,38.1)	2.5 (2,3)	59.5 (50.7,71)	41.7 (12.5,75)
Rednote	46.5 (42.8,50)	3.5 (3,3.5)	76.9 (71.8,86.3)	70.8 (50,87.5)
Bilibili	42 (33,47)	2.5 (1.5,3)	72.7 (68.2,80.3)	50 (50,100)
Kruskal–Wallis test: *p*	<0.001	<0.001	<0.001	<0.001
Pairwise comparisons: TikTok vs. Bilibili	<0.001	1.000	<0.001	0.001
Pairwise comparisons: TikTok vs. Rednote	<0.001	<0.001	<0.001	<0.001
Pairwise comparisons: Bilibili vs. Rednote	0.004	<0.001	0.196	<0.001

Data were presented as median (interquartile range). Differences among the three platforms were analyzed using the Kruskal–Wallis H test, followed by Bonferroni-adjusted pairwise comparisons. GQS, Global Quality Scale; PEMAT-U, the Patient Education Materials Assessment Tool-Understandability; PEMAT-A, the Patient Education Materials Assessment Tool-Actionability.

**Figure 2:**
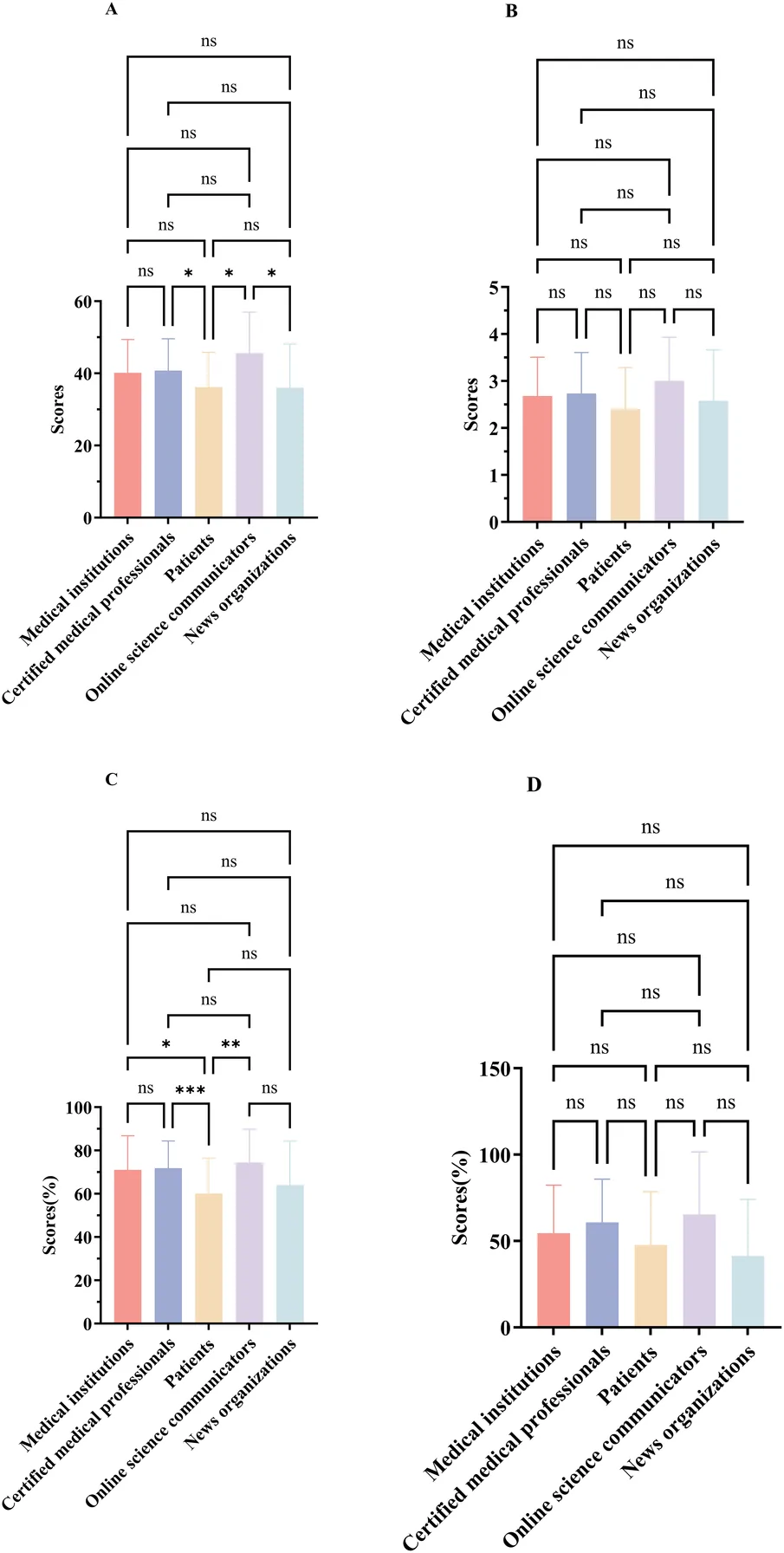
The DISCERN score, Global Quality Scale (GQS) score, Patient Education Materials Assessment Tool (PEMAT)–understandability, and PEMAT-actionability of videos on SLE from different sources. **(A)** the DISCERN score, **(B)** the GQS score, **(C)** PEMAT-U, and **(D)** PEMAT-A. ns, not significant. * *p* < 0.05, ** *p* < 0.01, *** *p* < 0.001.

Based on the analysis of the video sources, videos published by online science communicators received the highest scores across all evaluated indicators, followed by those from certified medical professionals. In contrast, videos posted by patients and news organizations received lower scores in all assessments. Notable disparities in DISCERN scores were identified between videos uploaded by certified medical professionals and those shared by patients (*p* < 0.05), between videos from patients and those from online science communicators (*p* < 0.05), and between videos from online science communicators and news organizations (*p* < 0.05). Regarding PEMAT-U scores, significant differences emerged between videos posted by medical institutions and those posted by patients (*p* < 0.05), between videos posted by patients and those by online science communicators (*p* < 0.01), and between videos posted by patients and those from certified medical professionals (*p* < 0.001). Conversely, no statistically significant differences were found in GQS scores or PEMAT-A scores across various publishers. See Figure 2 for details.

**Figure 3:**
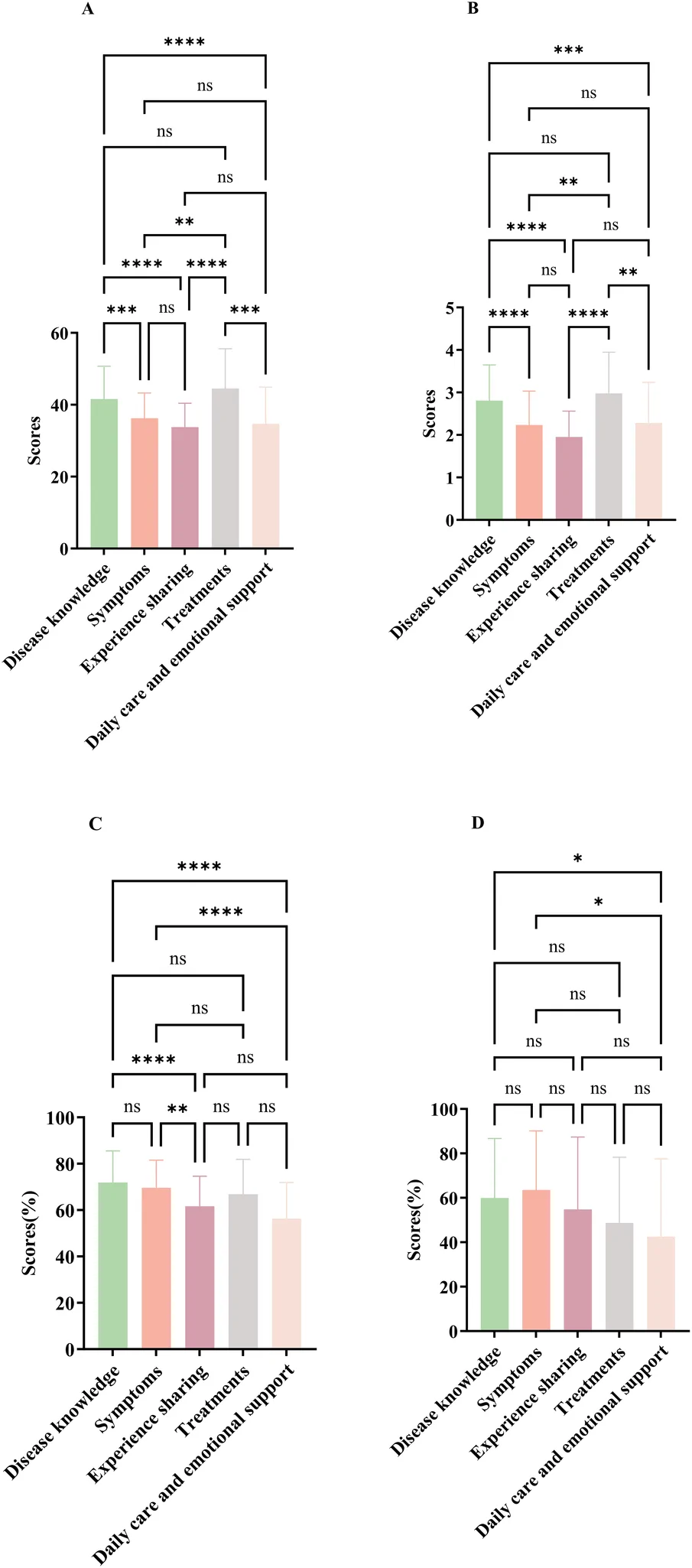
The DISCERN score, Global Quality Scale (GQS) score, Patient Education Materials Assessment Tool (PEMAT)–understandability, and PEMAT-actionability of videos on SLE from different categories. **(A)** the DISCERN score, **(B)** the GQS score, **(C)** PEMAT-U, and **(D)** PEMAT-A. ns, not significant. * *p* < 0.05, ** *p* < 0.01, *** *p* < 0.001, **** *p* < 0.0001.

This analysis categorizes the video content based on various scoring metrics. Among these metrics, the treatment category achieved the highest scores in both the DISCERN and GQS assessments, while the experience sharing category recorded the lowest scores. In contrast, the disease knowledge category received the highest PEMAT-U score, and the symptoms category obtained the highest PEMAT-A score. Conversely, the daily care and emotional support categories exhibited the lowest PEMAT scores. Significant disparities were observed between disease knowledge and various factors, including symptoms, experience sharing, daily care and emotional support, as well as between treatment modalities and these aforementioned factors, as indicated by both DISCERN and GQS scores (all *p* < 0.01). Notably, in terms of PEMAT-U scores, significant differences were evident between experience sharing and disease knowledge, symptoms (all *p* < 0.01), as well as daily care and emotional support relative to disease knowledge and symptoms (*p* < 0.0001). Furthermore, regarding PEMAT-A scores, significant differences were identified between daily care and emotional support and disease knowledge, symptoms (*p* < 0.05). See Figure 3 for details.

### Correlation analysis

This study examines the correlation between video interaction, dissemination data, and the quality and reliability of videos. The analysis reveals a significant positive correlation between video duration and both the DISCERN and GQS scores (*r* = 0.307 to 0.462, *p* < 0.001). Conversely, other video metrics—such as likes, collections, comments, and shares—demonstrated significant negative correlations with the DISCERN score, PEMAT-U score, and PEMAT-A score (*r* = −0.425 to −0.225, *p* < 0.001). Notably, no significant correlation was found between these metrics and the GQS score, as detailed in Table 3. Furthermore, Spearman's correlation analysis indicated significant positive correlations (*r* = 0.362 to 0.953, *p* < 0.01) among all variables, including video duration, likes, collections, comments, and shares, as shown in Table 4.

**Table 3:** The correlation analysis between video variables and the video quality.

Variables	DISCERN	GQS	PEMAT-U	PEMAT-A
Duration				
*r*	0.307**	0.462**	−0.008	0.092
*p*	<0.001	<0.001	0.915	0.157
Likes				
*r*	−0.225**^a^	0.023	−0.356**	−0.291**^a^
*p*	<0.001	0.784	<0.001	<0.001
Collections				
*r*	−0.300**	−0.116	−0.402**	−0.297**^a^
*p*	<0.001	0.055	<0.001	<0.001
Comments				
*r*	−0.305**	−0.088	−0.425**	−0.312**
*p*	<0.001	0.171	<0.001	<0.001
Shares				
*r*	−0.250**^a^	0.006	−0.352**	−0.284**^a^
*p*	<0.001	0.915	<0.001	<0.001

GQS, Global Quality Scale; PEMAT-U, the Patient Education Materials Assessment Tool-Understandability; PEMAT-A, the Patient Education Materials Assessment Tool-Actionability.

All reported *p*-values in this table have been adjusted for multiple comparisons using the Benjamini-Hochberg (BH) procedure to control the false discovery rate (FDR).

Correlation strength is classified as weak (|r| < 0.3), moderate (0.3 ≤ |r| < 0.5), or strong (|r| ≥ 0.5) following Cohen (1988).

a Weak correlation (|r| < 0.3); interpret with caution despite statistical significance.

** indicates *p* < 0.01 (both based on FDR-adjusted *p*-values).

**Table 4:** The correlation analysis between the video variables.

Variables	Duration	Likes	Collections	Comments	Shares
Duration					
*r*	1	0.415**	0.362**	0.380**	0.375**
*p*	-^a^	<0.001	<0.001	<0.001	<0.001
Likes					
*r*	0.415**	1	0.913**	0.936**	0.953**
*p*	<0.001	-	<0.001	<0.001	<0.001
Collections					
*r*	0.362**	0.913**	1	0.921**	0.892**
*p*	<0.001	<0.001	-	<0.001	<0.001
Comments					
*r*	0.380**	0.936**	0.921**	1	0.922**
*p*	<0.001	<0.001	<0.001	-	<0.001
Shares					
*r*	0.375**	0.953**	0.892**	0.922**	1
*p*	<0.001	<0.001	<0.001	<0.001	-

GQS, Global Quality Scale; PEMAT-U, the Patient Education Materials Assessment Tool-Understandability; PEMAT-A, the Patient Education Materials Assessment Tool-Actionability.

All reported *p*-values in this table have been adjusted for multiple comparisons using the Benjamini-Hochberg (BH) procedure to control the false discovery rate (FDR).

a Not applicable.

** indicates *p* < 0.01 (after FDR correction, all reported correlation coefficients remain statistically significant).

Spearman correlation analysis indicated that GQS and PEMAT-A scores on TikTok were negatively correlated with likes, collections, comments, and shares (*r* = −0.472 to −0.322, *p* < 0.01). Furthermore, the DISCERN and PEMAT-U scores revealed negative correlations with comments (*r* = −0.365 to −0.349, *p* < 0.01), with the DISCERN scores additionally showing a negative correlation with likes (*r* = −0.327, *p* < 0.01). In contrast, on Rednote, the GQS and PEMAT-U scores displayed negative correlations with collections and comments (*r* = −0.365 to −0.321, *p* < 0.01 to 0.05), while the PEMAT-U scores also exhibited a negative correlation with video duration (*r* = −0.452, *p* < 0.01). On Bilibili, the DISCERN, GQS, and PEMAT-U scores demonstrated positive correlations with video duration, likes, collections, and shares (*r* = 0.380 to 0.787, *p* < 0.01). See Table 5 for details.

**Table 5:** The correlation analysis between video variables and the video quality.

Variables	TikTok				Rednote				Bilibili			
	DISCERN	GQS	PEMAT-U	PEMAT-A	DISCERN	GQS	PEMAT-U	PEMAT-A	DISCERN	GQS	PEMAT-U	PEMAT-A
Duration												
* r*	0.241*^a^	0.224*^a^	−0.061	0.127	−0.173	0.007	−0.452**	0.166	0.694**	0.787**	0.425**	0.089
* p*	0.024	0.033	0.549	0.224	0.238	0.949	<0.001	0.148	<0.001	<0.001	<0.001	0.467
Likes												
* r*	−0.327**	−0.400**	−0.268*^a^	−0.462**	−0.154	−0.204	−0.295*^a^	0.070	0.626**	0.677**	0.380**	−0.031
* p*	0.001	<0.001	0.012	<0.001	0.278	0.150	0.030	0.724	<0.001	<0.001	<0.001	0.868
Collections												
* r*	−0.238*^a^	−0.322**	−0.187	−0.391**	−0.226*	−0.365**	−0.321**	−0.033	0.619**	0.658**	0.383**	−0.028
* p*	0.024	0.001	0.077	<0.001	0.106	0.006	0.016	0.818	<0.001	<0.001	<0.001	0.868
Comments												
* r*	−0.365**	−0.447**	−0.349**	−0.472**	−0.226*	−0.365**	−0.321**	−0.033	0.455**	0.500**	0.268**^a^	0.006
* p*	<0.001	<0.001	<0.001	<0.001	0.106	0.006	0.016	0.818	<0.001	<0.001	0.009	0.982
Shares												
* r*	−0.285*^a^	−0.328**	−0.181	−0.381**	−0.050	−0.102	−0.239*	−0.041	0.556**	0.612**	0.418**	−0.002
* p*	0.006	0.001	0.082	<0.001	0.818	0.540	0.105	0.818	<0.001	<0.001	<0.001	0.982

GQS, Global Quality Scale; PEMAT-U, the Patient Education Materials Assessment Tool-Understandability; PEMAT-A, the Patient Education Materials Assessment Tool-Actionability.

All reported *p*-values in this table have been adjusted for multiple comparisons using the Benjamini-Hochberg (BH) procedure to control the false discovery rate (FDR).

Correlation strength is classified as weak (|r| < 0.3), moderate (0.3 ≤ |r| < 0.5), or strong (|r| ≥ 0.5) following Cohen (1988).

a Weak correlation (|r| < 0.3); interpret with caution despite statistical significance.

* indicates *p* < 0.05, ** indicates *p* < 0.01.

## Discussion

This cross-platform evaluation of SLE-related videos revealed significant differences in duration, engagement, source, and thematic distribution. Bilibili exhibited a shorter median duration than TikTok and Rednote, yet with a far wider range (8–4850 s), accommodating both brief educational clips and extensive patient narratives. In contrast, TikTok and Rednote clustered around shorter formats, aligning with their emphasis on quick consumption. TikTok videos achieved the highest user interaction metrics, consistent with prior evidence that dissemination indicators reflect popularity (29–31), suggesting stronger public resonance on this platform.

The distribution of video sources was generally similar across platforms, with certified medical professionals and patients representing the predominant source categories. This pattern is consistent with previous studies reporting the coexistence of professionally generated health information and patient-shared experiences on online platforms (32, 33). Regarding thematic categories, “disease knowledge” was the core theme across all platforms, highlighting the central role of basic disease-related information in SLE health communication. Unlike studies of esophageal cancer that reported a higher proportion of “disease treatment” on Bilibili (32), a similar pattern was not observed in our study. This difference may be related to the distinct characteristics of SLE as a chronic autoimmune disease requiring individualized and long-term management, which may influence the types of health information prioritized in short-video content. Instead, TikTok and Rednote excelled in “treatment” and “daily care/emotional support,” highlighting complementary roles: Bilibili emphasizes foundational education, while the other platforms cater to practical daily and psychological needs.

Overall video quality and reliability were low to moderate across all platforms, consistent with findings from other disease areas (20, 34). Rednote demonstrated relatively higher scores on DISCERN, GQS, and PEMAT-related measures, whereas TikTok showed relatively lower scores across several quality assessments. However, even Rednote's median scores remained moderate, with reliability and comprehensibility rates of 70%–80%. These differences may be associated with platform-specific characteristics: Rednote's emphasis on high-quality practical information incentivizes robust content, whereas TikTok's entertainment-oriented, short-format nature may compromise medical depth and rigor (35).

Contrary to expectations based on prior reports (36, 37), videos created by online science communicators demonstrated higher DISCERN and PEMAT-U scores than videos from several other source categories. In contrast, patient- and media-generated videos generally showed lower scores across several quality-related measures. This finding may be related to the characteristics of science communication content, which often emphasizes structured information organization, visual presentation, and audience-oriented explanations. Although medical professionals indeed provide authoritative health information, variations in their communication styles and presentation approaches may influence the perceived quality of health-related videos (38, 39). Videos created by patients may offer valuable experiential perspectives; however, they may contain medical information that is less systematically organized compared to professionally produced materials. Notably, no significant differences were observed in the GQS and PEMAT-A scores across various types of publishers, suggesting that the overall quality and actionability of content may not depend solely on the identity of its creators. Additionally, disease knowledge videos scored highest, while experience-based videos scored lowest, underscoring the need for platform-specific quality enhancement strategies.

A pivotal discovery from this study was the disparate relationship between metrics related to video quality and user engagement across different platforms. Specifically, on TikTok and Rednote, several quality-related metrics exhibited inverse correlations with particular engagement indicators, implying that health information of superior quality does not invariably lead to heightened user engagement on these platforms. In contrast, on Bilibili, scores from DISCERN, GQS, and PEMAT-U demonstrated positive correlations with engagement indicators, while PEMAT-A scores did not show a statistically significant association. These platform-specific patterns might be attributed to variations in platform attributes, content ecosystems, and user preferences. Bilibili's content environment, which is relatively knowledge-centric, may be more conducive to educational health content and attract users with a stronger inclination towards learning (40). In contrast, the concise format and highly interactive nature of platforms like TikTok and Rednote may place a heightened emphasis on accessibility, emotional resonance, and audience engagement, potentially influencing the dissemination patterns of health-related content (41). Furthermore, variations in user preferences and content consumption behaviors across different platforms may contribute to disparities in how health information is received and interacted with (42).

Although this study focused on three algorithm-driven short-video platforms, other online health information channels—including AI-powered chatbots, long-form video platforms, and traditional health websites—differ fundamentally in their content delivery mechanisms and pose distinct quality challenges. Unlike short-video platforms that prioritize rapid, algorithmically curated dissemination, AI chatbots offer interactive, real-time responses but with variable reliability (43, 44); long-form platforms like YouTube allow in-depth exploration yet face similar quality-control deficits (44); and traditional text-based websites vary widely in readability and accuracy (8). Our selection of TikTok, Bilibili, and Rednote was motivated by their immense popularity, short-format algorithm-driven delivery, and predominance among younger demographics—features that create unique quality and reliability challenges warranting dedicated investigation. Future cross-platform comparative studies are needed to provide more comprehensive guidance for SLE patients and healthcare providers.

It is important to note that while short-video platforms show promise as supportive tools for facilitating access to health information, their content remains superficial compared with clinical guidelines in critical areas such as risk–benefit assessment, individualized recommendations, and recognition of warning signs. These platforms should therefore not serve as a primary source for patient information or independent clinical decision-making.

## Strengths and limitations

### Strengths of the study

A key innovation of this research is the inclusion of Rednote, a platform characterized by its active female user base. This inclusion enhances the sample's representativeness, considering the high prevalence of SLE among women, thereby addressing a research gap left by previous studies that have overlooked gender-specific platform characteristics.

### Limitations

Several limitations should be acknowledged. First, the cross-sectional design with a fixed search date prevented capturing dynamic content evolution; longitudinal studies are warranted. Second, the sampling strategy may have introduced selection bias, as ranking by “likes” could have overlooked high-quality new content, and the exclusion of videos with “insufficient health education content” relied on subjective judgment. Time-stratified sampling with objective selection criteria is recommended. Third, despite using validated instruments, scoring subjectivity remains unavoidable, and algorithmic confounding may have arisen from an uncleared search history. Blinded rating and private browsing with systematic history clearance should be adopted in future studies. Finally, the restriction to common disease names and Chinese-language videos on three short-video platforms limits generalizability. Cross-linguistic, cross-cultural, and cross-platform comparisons are needed to verify the robustness of these findings.

## Conclusion

Our findings demonstrate that the relationship between video quality and user engagement is platform- and evaluation measure-specific. On TikTok, several quality-related measures were negatively correlated with engagement indicators, with particularly consistent inverse associations observed for GQS and PEMAT-A. In contrast, on Bilibili, DISCERN, GQS, and PEMAT-U scores were positively correlated with engagement metrics, whereas PEMAT-A showed no significant association with engagement. Furthermore, videos created by online science communicators exhibited higher DISCERN and PEMAT-U scores than several other source categories, while no significant differences were observed in GQS or PEMAT-A. These findings highlight the importance of considering both content quality and audience engagement when developing strategies to improve the dissemination of reliable health information on short-video platforms. Future efforts should focus on integrating quality assessment into content evaluation strategies, establishing expert-informed review mechanisms, and promoting collaboration between healthcare professionals and science communicators. Regulatory bodies and relevant stakeholders may also consider developing multidimensional evaluation frameworks and training programs to support the creation and dissemination of high-quality health information.

## Data Availability

The original contributions presented in the study are included in the article/Supplementary Material, further inquiries can be directed to the corresponding authors.

## References

[1] Durcan L O'Dwyer T Petri M. Management strategies and future directions for systemic lupus erythematosus in adults. Lancet. (2019) 393(10188):2332–43. 10.1016/s0140-6736(19)30237-5 31180030

[2] Fava A Petri M. Systemic lupus erythematosus: diagnosis and clinical management. J Autoimmun. (2019) 96:1–13. 10.1016/j.jaut.2018.11.001 30448290 PMC6310637

[3] Siegel CH Sammaritano LR. Systemic lupus erythematosus: a review. JAMA. (2024) 331(17):1480–91. 10.1001/jama.2024.2315 38587826

[4] Guttmann A Denvir B Aringer M Buyon JP Belmont HM Sahl S. Evaluation of the EULAR/American College of Rheumatology classification criteria for systemic lupus erythematosus in a population-based registry. Arthritis Care Res (Hoboken). (2023) 75(5):1007–16. 10.1002/acr.24960 35638708 PMC11098446

[5] Tian J Zhang D Yao X Huang Y Lu Q. Global epidemiology of systemic lupus erythematosus: a comprehensive systematic analysis and modelling study. Ann Rheum Dis. (2023) 82(3):351–6. 10.1136/ard-2022-223035 36241363 PMC9933169

[6] Alduraibi FK Tsokos GC. Lupus nephritis biomarkers: a critical review. Int J Mol Sci. (2024) 25(2):805. 10.3390/ijms25020805 38255879 PMC10815779

[7] Bolouri N Akhtari M Farhadi E Mansouri R Faezi ST Jamshidi A. Role of the innate and adaptive immune responses in the pathogenesis of systemic lupus erythematosus. Inflamm Res. (2022) 71(5-6):537–54. 10.1007/s00011-022-01554-6 35298669

[8] Özduran E. YouTube As an information source for myofascial pain syndrome. In: Ünlü Z, editor. Current Approaches to Myofascial Pain Syndrome. 1st ed. Ankara: Türkiye Klinikleri (2026). p. 377–82. 10.5336/978-625-395-854-1_p377

[9] Özduran E Büyükçoban S. A content analysis of the reliability and quality of YouTube videos as a source of information on health-related post-COVID pain. PeerJ. (2022) 10:e14089. 10.7717/peerj.14089 36193427 PMC9526419

[10] Wanberg LJ Pearson DR. Evaluating the disease-related experiences of TikTok users with lupus erythematosus: qualitative and content analysis. JMIR Infodemiology. (2024) 4:e51211. 10.2196/51211 38631030 PMC11063877

[11] Chen J Wang Y. Social media use for health purposes: systematic review. J Med Internet Res. (2021) 23(5):e17917. 10.2196/17917 33978589 PMC8156131

[12] Hancı V Öner Ö Özduran E Gökel E. YouTube as a source of information about percutaneous tracheostomy. GMJ. (2023) 34:372–8. 10.12996/gmj.2023.77

[13] Özbek İC Özduran E Hancı V. Artificial intelligence in pain: a comprehensive review. Med J West Black Sea. (2026) 10(1):168–78. 10.29058/mjwbs.1881313

[14] Zheng S Tong X Wan D Hu C Hu Q Ke Q. Quality and reliability of liver cancer-related short Chinese videos on TikTok and Bilibili: cross-sectional content analysis study. J Med Internet Res. (2023) 25:e47210. 10.2196/47210 37405825 PMC10357314

[15] He Z Wang Z Song Y Liu Y Kang L Fang X. The reliability and quality of short videos as a source of dietary guidance for inflammatory bowel disease: cross-sectional study. J Med Internet Res. (2023) 25:e41518. 10.2196/41518 36757757 PMC9951074

[16] Swire-Thompson B Lazer D. Public health and online misinformation: challenges and recommendations. Annu Rev Public Health. (2020) 41:433–51. 10.1146/annurev-publhealth-040119-094127 31874069

[17] Özbek İC Hancı V Özduran E. Digital guidance: quality and readability analysis of artificial intelligence-generated spondyloarthropathy texts. Turk J Osteoporos. (2025) 31(1):12–8. 10.4274/tod.galenos.2024.76743

[18] Meunier B Jourde-Chiche N Mancini J Chekroun M Retornaz F Chiche L. Characteristics and information searched for by French patients with systemic lupus erythematosus: a web-community data-driven online survey. Lupus. (2016) 25(4):370–5. 10.1177/0961203315610644 26454067

[19] Zhang K Li Z Lin Y Guo Y Huang R Liu G. Bilibili, Douyin and Xiaohongshu as health information platforms for stroke: evaluating information quality and content. Sci Rep. (2025) 15(1):37705. 10.1038/s41598-025-21535-z 41152378 PMC12568935

[20] Sun K-X Cui C Mu F Tang M Gong R Yan Z. Reliability, quality, and science popularization of diabetes knowledge information in China video sharing platform: a cross-sectional study. Digit Health. (2025) 11:20552076251393298. 10.1177/20552076251393298 41181547 PMC12576113

[21] Zuo Q-H Du K Li A Zhang C-Y Guo R Chen P. Quality and reliability of osteoarthritis-related health information on short video platforms: a cross-sectional comparative study of TikTok and Bilibili. Front Digit Health. (2025) 7:1623247. 10.3389/fdgth.2025.1623247 41281294 PMC12634650

[22] Qin Z Ng S Wu W Zhang S. What Chinese women seek in mental health apps: insights from analyzing Xiaohongshu user posts during the COVID-19 pandemic. Healthcare (Basel. (2024) 12(13):1297. 10.3390/healthcare12131297 38998832 PMC11241336

[23] Jiang S Sun H Chen X. Assessment and analysis of content and quality of children's influenza vaccine information on BiliBili, Douyin, and Xiaohongshu. Front Digit Health. (2026) 7:1591347. 10.3389/fdgth.2025.1591347 41696693 PMC12902773

[24] Charnock D Shepperd S Needham G Gann R. DISCERN: an instrument for judging the quality of written consumer health information on treatment choices. J Epidemiol Community Health. (1999) 53(2):105. 10.1136/jech.53.2.105 10396471 PMC1756830

[25] Shan Y Xing Z Dong Z Ji M Wang D Cao X. Translating and adapting the DISCERN instrument into a simplified Chinese version and validating its reliability: development and usability study. J Med Internet Res. (2023) 25:e40733. 10.2196/40733 36729573 PMC9936359

[26] Du RC Zhang Y Wang MH Lu NH Hu Y. Tiktok and Bilibili as sources of information on Helicobacter pylori in China: a content and quality analysis. Helicobacter. (2023) 28(5):e13007. 10.1111/hel.13007 37452727

[27] Shuab A Krakowiak M Szmuda T. The quality and reliability analysis of YouTube videos about insulin resistance. Int J Med Inform. (2023) 170:104960. 10.1016/j.ijmedinf.2023.104987 36525801

[28] Shoemaker SJ Wolf MS Brach C. Development of the patient education materials assessment tool (PEMAT): a new measure of understandability and actionability for print and audiovisual patient information. Patient Educ Couns. (2014) 96(3):395–403. 10.1016/j.pec.2014.05.027 24973195 PMC5085258

[29] Zhu F Jiao J Huang Y Xiao F Zuo W Chen M. A preliminary study of the surgical approach for posterior tibial plateau fractures: based on posterior fragment segment classification. Injury. (2022) 53(11):3820–7. 10.1016/j.injury.2022.09.009 36116959

[30] Guan J-L Xia S-H Zhao K Feng L-N Han Y-Y Li J-Y. Videos in short-video sharing platforms as sources of information on colorectal polyps: cross-sectional content analysis study. J Med Internet Res. (2024) 26:e51655. 10.2196/51655 39470708 PMC11558218

[31] Liang M Yang F Lu S Zhu C Wang J Yan W. Quality and reliability of prostate cancer-videos on TikTok and Bilibili: cross-sectional content analysis study. Digit Health. (2025) 11:20552076251376263. 10.1177/20552076251376263 40918078 PMC12409058

[32] Zhu W He B Wang X Du Y Young K Jiang S. Information quality of videos related to esophageal cancer on Tiktok, Kwai, and Bilibili: a cross-sectional study. BMC Public Health. (2025) 25(1):2245. 10.1186/s12889-025-23475-9 40604838 PMC12220660

[33] Yang Y Jiang S Ning X Xie L Zhang X Yu L. Assessing the video content quality of TikTok and Bilibili as health information sources for systemic lupus erythematosus: a cross-sectional study. Int J Rheum Dis. (2025) 28(6):e70341. 10.1111/1756-185x.70341 40551617

[34] Cai QY Tang J Meng SZ Sun Y Lan X Liu TH. Quality assessment of videos on social media platforms related to gestational diabetes mellitus in China: a cross-section study. Heliyon. (2024) 10(7):e29020. 10.1016/j.heliyon.2024.e29020 38617917 PMC11015130

[35] Niu Z Hao Y Yang F Jiang Q Jiang Y Zhang S. Quality of pancreatic neuroendocrine tumor videos available on TikTok and Bilibili: content analysis. JMIR Form Res. (2024) 8:e60033. 10.2196/60033 39661988 PMC11655045

[36] Chen Y Wang Q Huang X Zhang Y Li Y Ni T. The quality and reliability of short videos about thyroid nodules on BiliBili and TikTok: cross-sectional study. Digit Health. (2024) 10:20552076241288831. 10.1177/20552076241288831 39381823 PMC11459542

[37] D'Ambrosi R Hewett TE. Validity of material related to the anterior cruciate ligament on TikTok. Orthop J Sports Med. (2024) 12(2):23259671241228543. 10.1177/23259671241228543 38405012 PMC10893838

[38] Mueller SM Hongler VNS Jungo P Cajacob L Schwegler S Steveling EH. Fiction, falsehoods, and few facts: cross-sectional study on the content-related quality of atopic eczema-related videos on YouTube. J Med Internet Res. (2020) 22(4):e15599. 10.2196/15599 32329744 PMC7210495

[39] Shortt MT Berg SH Wiig S Lungu DA Smeets I Thune H. Exploring health experts’ and creative communicators' focus in pandemic video communication: a qualitative study. Front Commun. (2022) 7:886768. 10.3389/fcomm.2022.886768

[40] Prokop-Dorner A Zając J Bała MM. Communicating evidence-based information on the effects of health interventions to various types of recipients - a qualitative study on the perception of formats of information among lay and professional audiences. Ann Agric Environ Med. (2024) 31(4):529–45. 10.26444/aaem/186920 39743712

[41] Shao C Ciampaglia GL Varol O Yang KC Flammini A Menczer F. The spread of low-credibility content by social bots. Nat Commun. (2018) 9(1):4787. 10.1038/s41467-018-06930-7 30459415 PMC6246561

[42] Park N Kee KF Valenzuela S. Being immersed in social networking environment: Facebook groups, uses and gratifications, and social outcomes. Cyberpsychol Behav. (2009) 12(6):729–33. 10.1089/cpb.2009.0003 19619037

[43] Kara M Özduran E Kara MM Özbek İC Hancı V. Evaluating the readability, quality, and reliability of responses generated by ChatGPT, Gemini, and Perplexity on the most commonly asked questions about ankylosing spondylitis. PLoS One. (2025) 20(6):e0326351. 10.1371/journal.pone.0326351 40531978 PMC12176213

[44] Özduran E Hancı V. Evaluating the readability, quality, and reliability of online information on Sjögren's syndrome. Indian J Rheumatol. (2023) 18:16–25. 10.4103/injr.injr_56_22

