# Correction: Quality and reliability assessment of systemic lupus erythematosus related health information on short video platforms

**DOI:** 10.3389/fdgth.2026.1970952

**Published:** 2026-09-03

**Authors:** Lingling Gao, Yujie Yang, Yuchen Wang, Kunling Wang, Wanqing Li, Hongrui Wen, Yunan Zhang, Xiangfeng Li, Wei Bai

**Affiliations:** 1 Peking Union Medical College Hospital (CAMS), Beijing, China; 2 Chinese Academy of Medical Sciences and Peking Union Medical College, Beijing, China

**Keywords:** health information, quality assessment, short video, social media, systemic lupus erythematosus

Authors Yujie Yang, Yuchen Wang and Lingling Gao were erroneously omitted as equal contributing authors.

The original version of this article has been updated.

